# Identification of *ATP1A3* Mutations by Exome Sequencing as the Cause of Alternating Hemiplegia of Childhood in Japanese Patients

**DOI:** 10.1371/journal.pone.0056120

**Published:** 2013-02-08

**Authors:** Atsushi Ishii, Yoshiaki Saito, Jun Mitsui, Hiroyuki Ishiura, Jun Yoshimura, Hidee Arai, Sumimasa Yamashita, Sadami Kimura, Hirokazu Oguni, Shinichi Morishita, Shoji Tsuji, Masayuki Sasaki, Shinichi Hirose

**Affiliations:** 1 Department of Pediatrics, School of Medicine, Fukuoka University, Fukuoka, Japan; 2 Central Research Institute for the Molecular Pathomechanisms of Epilepsy, Fukuoka University, Fukuoka, Japan; 3 Department of Child Neurology, National Center of Neurology and Psychiatry, Kodaira, Japan; 4 Department of Neurology, Graduate School of Medicine, The University of Tokyo, Tokyo, Japan; 5 Department of Computational Biology, Graduate School of Frontier Sciences, The University of Tokyo, Kashiwa, Japan; 6 Department of Neurology, Chiba Children's Hospital, Chiba, Japan; 7 Division of Child Neurology, Kanagawa Children's Medical Center, Yokohama, Japan; 8 Division of Child Neurology, Osaka Medical Center and Research Institute for Maternal and Child Health, Izumi, Japan; 9 Department of Pediatrics, Tokyo Women's Medical University, Tokyo, Japan; University of Thessaly, Greece

## Abstract

**Background:**

Alternating hemiplegia of childhood (AHC) is a rare disorder characterized by transient repeated attacks of paresis and cognitive impairment. Recent studies from the U.S. and Europe have described *ATP1A3* mutations in AHC. However, the genotype-phenotype relationship remains unclear. The purpose of this study was to identify the genetic abnormality in a Japanese cohort of AHC using exome analysis.

**Principal Findings:**

A total of 712,558 genetic single nucleotide variations in 8 patients with sporadic AHC were found. After a series of exclusions, mutations of three genes were regarded as candidate causes of AHC. Each patient harbored a heterozygous missense mutation of *ATP1A3*, which included G755C, E815K, C927Y and D801N. All mutations were at highly conserved amino acid residues and deduced to affect ATPase activity of the corresponding ATP pump, the product of *ATP1A3*. They were *de novo* mutations and not identified in 96 healthy volunteers. Using Sanger sequencing, E815K was found in two other sporadic cases of AHC. In this study, E815K was found in 5 of 10 patients (50%), a prevalence higher than that reported in two recent studies [19 of 82 (23%) and 7 of 24 (29%)]. Furthermore, the clinical data of the affected individuals indicated that E815K resulted in a severer phenotype compared with other *ATP1A3* mutations.

**Interpretation:**

Heterozygous *de novo* mutations of *ATP1A3* were identified in all Japanese patients with AHC examined in this study, confirming that *ATP1A3* mutation is the cause of AHC.

## Introduction

Alternating hemiplegia of childhood (AHC) (MIM 104290) is a rare disorder characterized by transient repeated attacks of paresis on either one or both sides of the body, occulomotor and autonomic abnormalities, movement disorders, and cognitive impairment [1], [2]. AHC is predominantly observed in sporadic cases without familial history, although familial AHC with autosomal dominant inheritance has also been reported [3]. Only about 50 patients with sporadic AHC have been reported in Japan and the estimated prevalence of AHC is one in a million births [4].

Since the clinical features of AHC share similarity with those of familial hemiplegic migraine (FHM), previous studies applied mutational analyses of *CACNA1A* (NM_000068) and *ATP1A2* (MN_000702), which are responsible for two types of FHM, FHM1 (MIM 601011) [5] and FHM2 (MIM 182340) [6], [7], respectively, to explore the genetic cause of AHC. Although T378N, a mutation of *ATP1A2*, was identified in four affected members of a Greek family with familial AHC [3], mutations of *ATP1A2* have neither been observed in other familial cases nor in sporadic cases of AHC. Thus, candidate gene approaches have been unsuccessful in identifying the molecular pathogenic mechanism of AHC.

**Figure 1:**
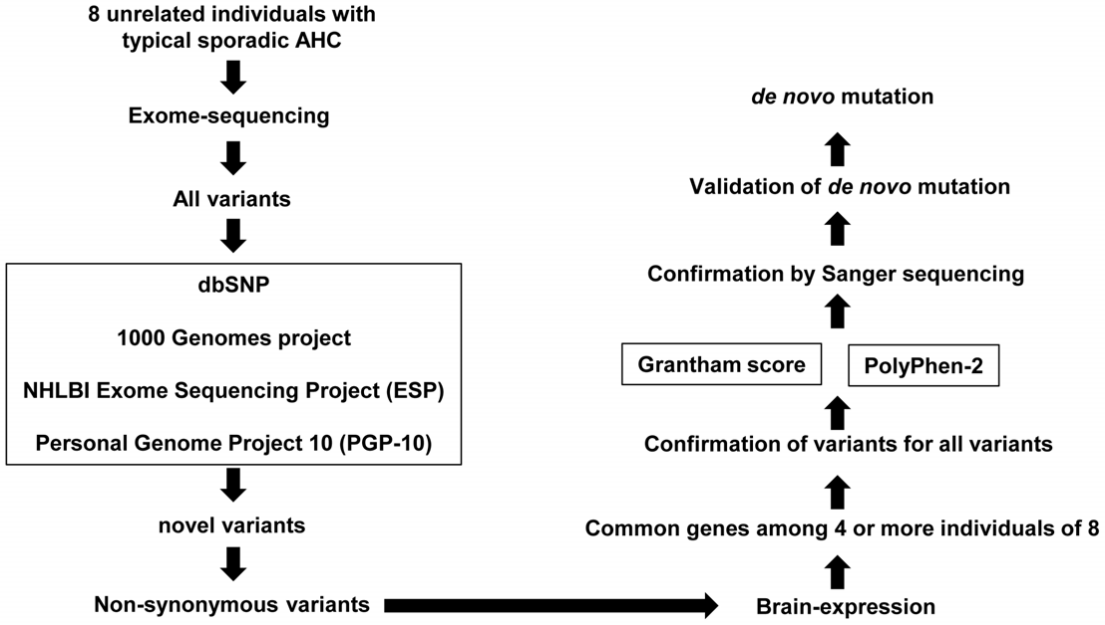
Pipeline for detection of novel *de nov*o mutations. The pipeline was used to identify pathogenic mutations of alternating hemiplegia of childhood (AHC). All genetic variants detected by exome sequencing are sequentially filtered through the pipeline. First, variations are screened according to databases of registered single nucleotide polymorphisms (SNP) and only non-registered SNP undergo the next selection as “Novel variants”. In the next step, non-synonymous novel variants of genes expressed in the central nervous system are selected. When variations of the same gene are found in the patient, the impact of such variation is evaluated *in silico* using Grantham score and PolyPhen-2. Mutations identified at this stage are reconfirmed by Sanger sequence. *De novo* mutation is validated by analyzing samples from parents. Mutations considered pathogenic are sought in other patients with AHC if necessary.

To elucidate the molecular basis of AHC, we hypothesized that sporadic AHC is caused by *de novo* mutations among novel non-synonymous coding variants, which are shared in patients with AHC. To test this hypothesis, we built a *de nov*o mutation detection pipeline using the exome sequencing method (Figure 1). Using this technique, we found that *de novo* mutations of *ATP1A3* (NM_152296) cause sporadic AHC.

## Results

A total of 712,558 genetic single nucleotide variations (SNVs) and 141,933 small indels were found, including previously known and synonymous genomic variations (Table 1). The ratios of non-overlapping variations in these patients are comparable to those of Asian or Japanese populations (Figure S1). The candidate variants were selected in the following processes based on the pipeline designed in the present study (Figure 1).

**Table 1:** Distribution of novel non-synonymous single nucleotide polymorphisms including brain-expressed genes in eight patients with AHC.

Patient ID	Total		Novel				
	Variant	Gene	Variant	Variant (NS/SS)	Gene (NS/SS)	Brain expressed variant (NS/SS)	Brain expressed gene (NS/SS)
I-1	229,647	5,590	6,195	282	270	77	75
II-1	200,443	5,656	5,934	316	299	86	82
III-1	125,855	5,489	4,304	342	327	100	93
IV-1	251,550	5,701	7,568	405	376	129	118
V-1	174,045	5,503	6,251	323	302	95	91
VI-1	231,603	5,744	6,785	402	388	111	108
VII-1	177,446	5,613	5,344	330	313	101	96
VIII-1	178,175	5,608	4,767	295	282	78	77
Total	712,558	1,3517	39,414	2,449	2,131	718	630

NS: non-synonymous variants, SS: splice-site acceptor/donor variants.

To select variants as candidate mutations for AHC, variations that are registered in the genomic variation databases were excluded, which resulted in a total of 39,414 single nucleotide variants and 48,056 indels. The next step was designed to select non-synonymous coding variations and those affecting splice sites, which resulted in the identification of 2,449 variations in 2,131 genes and 246 indels in 232 genes.

We then selected variations in genes expressed in the central nervous system (CNS) (Note S1) [8]. Using this filter, we further narrowed the list to 718 non-synonymous SNVs and 76 indels (Table 1). We then identified variations that were frequently shared among the 8 patients with sporadic AHC. We found that six patients (II-1, III-1, IV-1, VI-1, VII-1, and VIII-1) carried a common variant (c.2813T>G: V938G) of *CNTN4*, four patients carried heterozygous variants of *SYNE1* (c.3955G>A: E1319K in VII-1, c.7196T>G: V2399A in III-1, c.10126A>G: M3376V in V-1, and c.24665G>A: R8222Q in I-1) and five patients carried heterozygous variants (c.2263G>T: G755C, c.2443G>A: E815K, and c.2780G>A: C927Y) of *ATP1A3* (Table 2). These variations were then subjected to validation by Sanger sequencing. The SNV of c.2813T>G of *CNTN4* was not confirmed by Sanger sequencing, indicating that it is an error of exome sequencing.

**Table 2:** *ATP1A3* variants found in eight individuals with AHC.

Patient	Chromosome (position)	Exon	SNV	Amino acid change
I-1	19 (42479781)	16	c. 2263 G>T	G755C
II-1	19 (42474436)	18	c. 2443 G>A	E815K
III-1	19 (42474436)	18	c. 2443 G>A	E815K
IV-1	19 (42474436)	18	c. 2443 G>A	E815K
V-1	19 (42472976)	20	c. 2780 G>A	C927Y
VI-1	19 (42474557)	17	c. 2401 G>A	*D801N
VII-1	19 (42474557)	17	c. 2401 G>A	*D801N
VIII-1	19 (42474557)	17	c. 2401 G>A	*D801N

SNV: single nucleotide variation,

* D801N was initially not considered a novel mutation but confirmed later by re-analysis.

We then sought all non-synonymous coding variants of *SYNE1* in all variants identified by exome sequencing regardless of whether they were novel or had been reported previously. A total of 19 non-synonymous coding SNVs (10 in I-1, 10 in II-1, 8 in III-1, 10 in IV-1, 9 in V-1, 8 in VI-1, 10 in VII-1, and 9 in VIII-1) were found in 8 patients. Sanger sequencing was performed to search for the 4 novel variants, which were found in the 4 patients, in 96 controls and parents of the 4 patients. Among the novel variants, E1319K, V2399A and M3376V of *SYNE1* were found in 2, 2 and 2 individuals of the 96 controls, respectively. R8222Q was not found in the control. However, each of the 4 variants including R8222Q was inherited from one of the healthy parents of the probands. Taken together, these results suggest that *SYNE1* is unlikely to be the gene responsible for AHC.

Three heterozygous variants (c.2263G>T: G755C, c.2443G>A: E815K, and c.2780G>A: C927Y) of *ATP1A3* were found in 5 of the 8 patients (Table 2). We then reviewed the data of exome analysis, with a special focus on *ATP1A3*, and found another variant (c.2401G>A: D801N) in the other 3 patients. The D801N was not initially classified as a novel variant through our pipeline, since a variant involving D801 had already been registered (though the mutation was D801Y). The D801Y mutation was reported to cause rapid-onset dystonia-parkinsonism (RDP/DYT12) (MIM 128235) [9].

**Figure 2:**
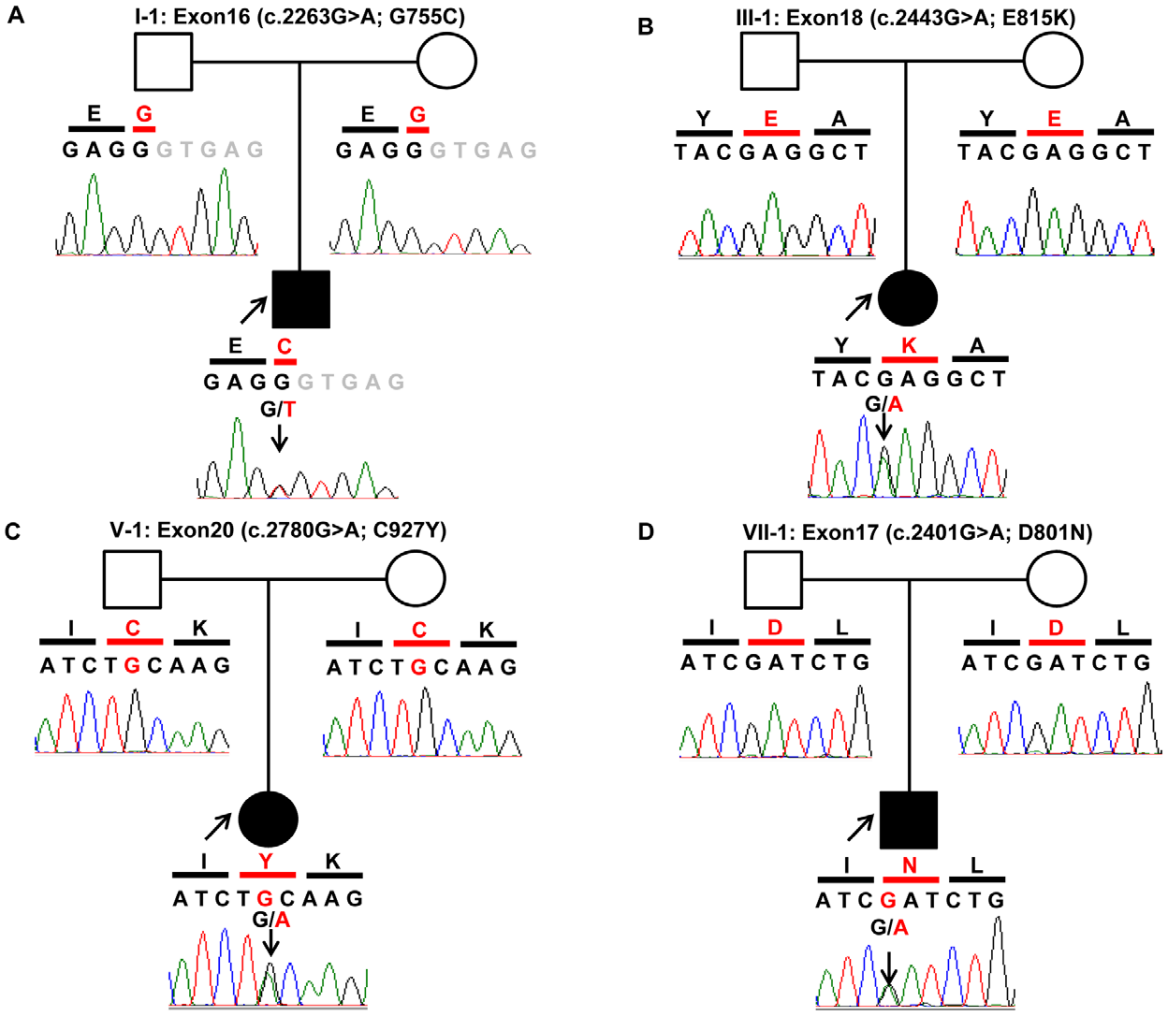
Chromatograms of four *de novo* mutations identified in *ATP1A3*. Data were obtained by Sanger sequencing during the confirmation process. In trio of each pedigree, black shadow represents the proband. In the chromatograms, *Black letters* show exonic nucleotide sequences, *gray letters* show intronic nucleotide sequences. Amino acids are shown in a single letter notation. Nucleotides and amino acids in red indicate mutations. (A) G755C was identified only in Patient I-1. (B) E815K was identified in Patients II-1, III-1, IV-1, IX-1 and X-1. (C) C927Y was identified in Patient V-1 only. (D) D801N was identified in Patients VI-1, VII-1 and VIII-1. None of the mutations was detected in the father or mother except for Patient IX-1, whose parents refused to undergo genetic analysis.

Sanger sequencing of *ATP1A3* confirmed four heterozygous mutations; D801N mutation in Patients I-1, VI-1 and VII-1, G755C mutation in Patient II-1, E815K in Patients III-1, IV-1 and V-1, and C927Y mutation in Patient VIII-1 (Figure 2). None of the variants were detected in the parents of each patient, indicating that these mutations were *de novo*. None of these variants was detected in any of the 96 healthy subjects.

Sanger sequence analysis for *ATP1A3* was further conducted in two other unrelated individuals with sporadic AHC (Patients IX-1 and X-1, Table 3). The analysis identified a heterozygous E815K in both patients while neither of the parents of these two patients had the mutation, confirming that the mutation was also *de novo*. These findings in the two patients provided compelling evidence for the pathogenic role of *ATP1A3* mutation in sporadic AHC. Taken together, we identified a total of four *ATP1A3* mutations in the 10 patients studied and these *de novo* mutations were considered pathogenic mutations involved in the etiology of AHC.

**Table 3:** Clinical data of 10 unrelated individuals with AHC.

Patient ID	I-1	II-1	III-1	IV-1	V-1	VI-1	VII-1	VIII-1	IX-1	X-1
Mutations	G755C	E815K	E815K	E815K	C927Y	D801N	D801N	D801N	E815K	E815K
Age (year)/sex	18/male	13/male	32/female	6/male	16/female	17/male	9/male	12/male	9/male	1/male
Age at onset (day)	60	17	2	1	60	1	120	0	Infant	Neonatal
Age at onset of paralysis (month)	6	10	12	4	12	4	9	9	Infant	9
Initial symptoms/signs	L versive movement of neck, monocular deviation of L eye to the left	Tonic fits	Tonic fits	Upward gaze, tonic fits	Nystagmus, ocular deviation to right	Nystagmus, focal clonic seizure	Clonic seizure	Nystagmus	Apnea	Nystagmus, downward gaze, tonic fits
Paralytic type	Flaccid	Flaccid	Flaccid	Flaccid	Rigid	Flaccid	Flaccid	Flaccid	Flaccid	Flaccid
Paralytic symptoms	Paralysis of unilateral arm or leg on R or L, or hemiparesis, sometimes continues with shift to opposite side. Rarely quadriplegia.	Paralysis of unilateral arm or leg on R or L, or hemiparesis, sometimes shifts to opposite side. Rarely quadriplegia.	Hemiparesis. Sometimes quadriplegia. No episodic paralysis since stabilizing of quadriplegia at 14 years.	Paralysis or hemiparesis of R arm.	Rigidity of R arm. Alternating flaccid hemiplegia since 1 year of age.	Alternating hemiparesis every 2–3 months	Alternating hemiplegia (R>L), only a few days every month.	R or L unilateral arm or leg paralysis, sometimes systemic paralysis. Tendency to occur following tonic fits.	Quadriplegia with/without bulbar palsy, for a few min to several hrs every day. Sometimes hemiplegia. Sometimes paralysis shifts to other parts.	Exterior ocular deviation on R side. Systemic cataplexy. Alternating paraparesis
Other neurological abnormalities	Choreoathetosis, aphonia	Choreoathetosis, facial dyskinesia	Dystonia, oral or facial dyskinesia	Aphonia	Spastic diplegia	None	Left hemidystonia	Dystonia	Dystonia	Head lag, nystagmus, ocular deviation
Motor development	walks alone	stands with support	walks with support	sits alone	walks alone	walks alone	walks alone	walks with support	Unable to sit	rolling over
Intellectual development	two words	only words	only words	no words	Normal	three phrases	three word phrases	only words	No words	delay
Regression	No	Yes	Yes	No	Yes	No	No	Yes	Yes	No
Epilepsy	4 years	2 years	4 years	None	None	None	4months	8 years	Yes	9months
Epileptic status	No	Yes	Yes	No	No	No	No	Yes	Yes	Yes
Headache	Yes	Yes	No	No	No	No	No	No	unknown	unknown
Head MRI	Normal	Cerebellar atrophy	Cerebellar atrophy	Normal	Mild enlargement of inferior horns bilaterally	Normal	Normal	High intensity in hippocampus	N/A	Normal
Respiratory status	Apnea	Normal	Use of ventilator	Apnea	Normal	Normal	Normal	Apnea	Apnea	Apnea
Effective drugs for paralysis	flunarizine	CZP	CZP, flunarizine	flunarizine	CZP	flunarizine	flunarizine	flunarizine	none (flunarizine not tried)	MDL
Family history	None	None	None	Headache, epilepsy	None	None	Migraine	Headache, epilepsy	Headache	None
Gestational age	40 weeks	34 weeks 3days	42 weeks	40 weeks	unknown	41 weeks 4 days	39 weeks 3 days	41 weeks	40 weeks	37 weeks 3 days
Birth weight (g)	3148	2218	3260	3392	unknown	3526	3200	3008	3550	2962
Asphyxia	None	No crying unless stimulated	Unknown	None	unknown	None	unknown	None	None	None

MDL: midazolam, CZP: clonazepam, L: left, R: right.

The clinical features of AHC patients with *de novo* mutations are summarized in Table 3. Four of the 5 patients with E815K and 1 of the 3 patients with D801N had respiratory abnormalities such as apnea, and one of the patients with E815K required mechanical ventilation. Furthermore, patients with E815K and D801N suffered from status epilepticus, and various involuntary movements were encountered in those harboring E815K mutation. Unfortunately, the small number of patients in our study precluded any firm conclusions backed by proper statistical analysis between genotype and phenotype. However, the results suggested the frequent presence of severe neurological complications, such as aphonia, choreoathetosis, dyskinesia and epilepsy, in individuals with E815K (Table 3). The attending physicians also provided answers to our survey on medications that were considered effective in the control of paralysis (Table 3).

## Discussion

By applying the exome sequencing strategy, we have demonstrated in the present study that *de novo ATP1A3* mutations cause sporadic AHC. Our work provides evidence that *ATP1A3* is the responsible gene for sporadic AHC, a rare but devastating disease that lacks proper treatment so far. At the time of the writing of this communication, two independent research groups, one from the USA and the other from Germany [10], [11], reported similar findings. Collectively, the three studies confirm that *ATP1A3* is the causative gene for AHC.

*ATP1A3* is a member of the gene family that encodes the alpha subunits of Na^+^/K^+^ transporting ATPase, which regulates the electrochemical gradients of Na^+^ and K^+^ through active transport. These ions are essential for regulation of cellular osmolality and the action potentials of excitable membrane. *ATP1A1*, *ATP1A2* and *ATP1A3* encode alpha 1, 2 and 3 subunits, respectively, which are mainly expressed in interneurons and pyramidal cells[12], suggesting that they play important roles in the brain.

**Figure 3:**
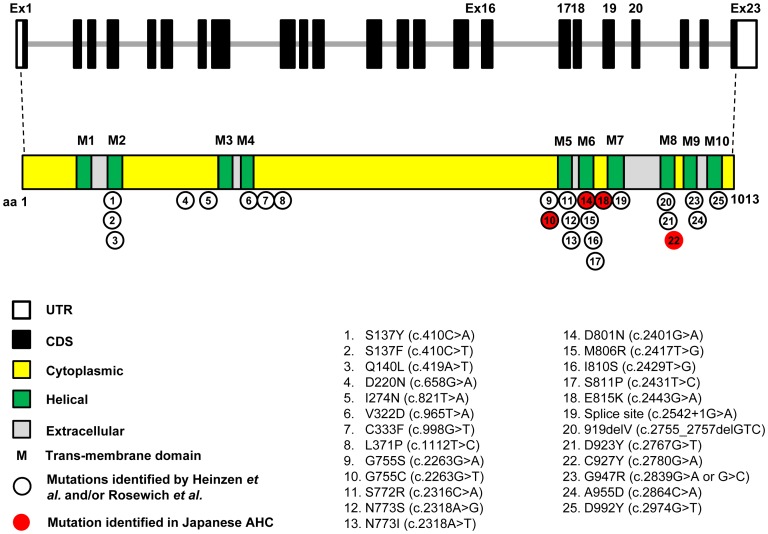
*ATP1A3* mutations and their protein domain structures. *Black lined circle:* Mutations reported recently [10], [11]. *Red colored circle:* Mutations identified in the present study in a Japanese cohort with AHC. The *ATP1A3* gene consists of 23 exons that encode several domains in the ATP1A3 protein molecule, including 6 cytoplasmic, 10 helical and 5 extracellular domains. G755C and E815K were located in the cytoplasmic domains. Notably, E815K was resident of the transmembrane domain rather than the cytoplasmic domain. D801N and C927Y were located in the helical domains. C927Y was identified in this study only and hence considered novel.

A total of 25 mutations identified to date reside in or near transmembrane domains (Figure 3). The G755C and E815K are at the cytoplasmic domain. However, E815K resides more in the transmembrane domain than in the cytoplasmic domain. The D801N and C927Y are at the transmembrane domains, M6 and M8, respectively, and form a helical structure. Also, C927Y identified in our study is a novel mutation.

**Figure 4:**
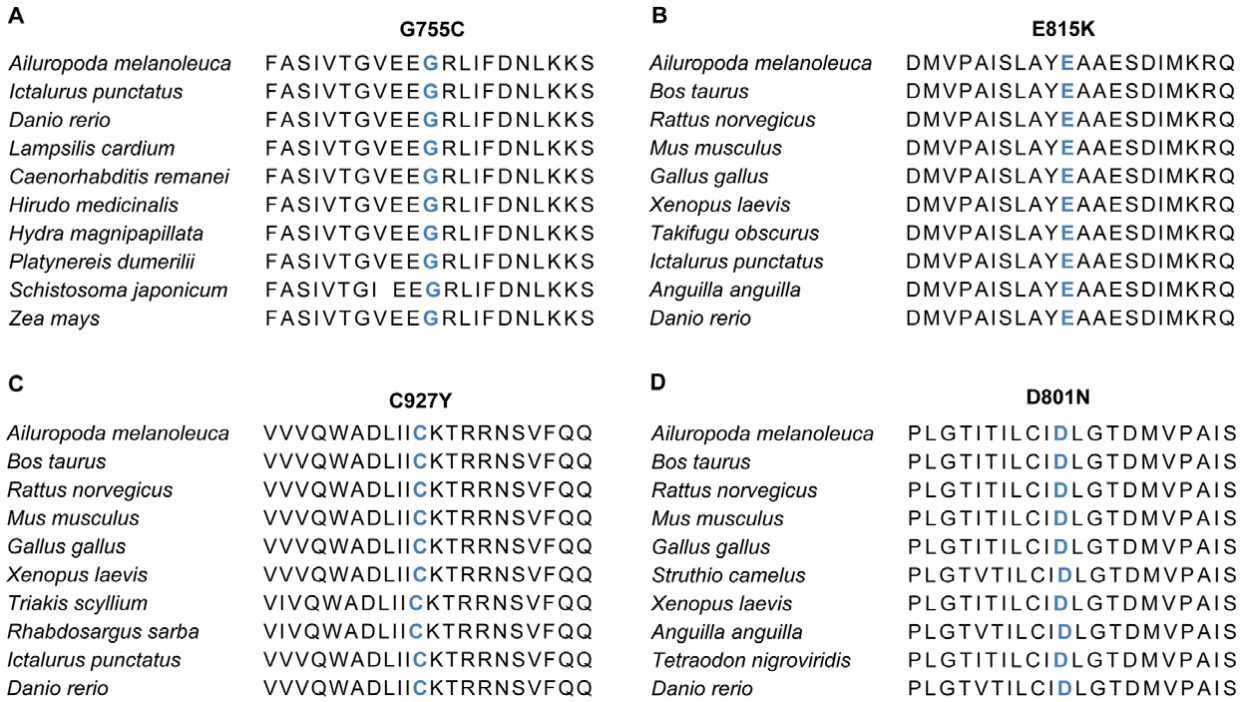
Homologous comparison of altering-protein. *Blue letters:* altering-protein by mutation, *red letters:* differential protein with human. (A) G755C changed by novel SNVs (c.2263G>T) of *ATP1A3* in Patient I-1. (B) E815K changed by novel SNVs (c.2443 G>A) of *ATP1A3* in Patients II-1, III-1, IV-1, IX-1 and X-1. (C) C927Y changed by novel SNVs (c.2780 G>A) of *ATP1A3* in Patient V-1. (D) D801N changed by novel SNVs (c.2401 G>A) of *ATP1A3* in Patient VI-1, VII-1 and VIII-1.

The amino acids substituted in each mutation are highly conserved among Na^+^/K^+^ ATPase isoforms of various species (Figure 4), suggesting that the amino acids are crucial for ATPase function. In fact, *in silico* analysis of the mutations identified in the present study suggests a profound damage of the ATPase molecule and hence accord well with functional deficits of the ATPase encountered with the recently described mutations [10].

It is noteworthy that several mutations of *ATP1A3* have been reported to cause RDP [9]. RDP is an autosomal dominant disease characterized by abrupt onset of dystonia and Parkinsonism, developing within minutes to days of onset [13]–[16]. Recently reported were two infantile RDP patients with *ATP1A3* mutations (R756H and D923N); onset began for one of them at 11 months and for the other at 4 years of age. Major symptoms included motor delay, hypotonia, and ataxia [17], [18]. Involuntary movements such as dystonia overlap with AHC, however, their clinical features and age of onset are different than those of AHC, which mainly shows repeated attacks of alternating hemiplegia and which begins with abnormal ocular movements by 3 months of age. Both typical and infantile RDP show different clinical features and processes than AHC, although *ATP1A3* seems to be pathologically involved in both disorders. In particular, D801N, one of the *ATP1A3* mutations identified in the present study, affected D801, where D801Y had been found in RDP. Thus, it seems that two substitutions in the same amino acid result in two distinguished phenotypes. Initially, we could not identify D801N in *ATP1A3* from novel variant. The reason for the erroneous results was the extraction of novel variants from all the variants using chromosome position only during the collation of databases. The position 42474557 of chromosome 19, where the G to A transversion resulted in D801N identified by our exome sequencing, had been registered as the nucleotide where the G to T transition is identified in rapid-onset dystonia-parkinsonism. Based on the backup plans involving reconfirmation of the gene identified with novel variants, using all variants, and to re-sequence the gene in our pipeline with the Sanger sequencer, D801N was not overlooked in the present study. These results suggest that confirmation by Sanger sequencer is useful in avoiding any oversight in the field of gene identification.

Functional analysis of *ATP1A3* mutations in RDP by haplo-insufficiency demonstrated low protein levels of the corresponding ATPase [9]. In addition, Heinzen *et al.* demonstrated that none of the mutations causes AHC reduced protein levels, whereas both mutations of AHC and those of RDP reduced ATPase activity [10]. These studies suggested that mutations identified in AHC affect the Na^+^/K^+^ ATPase pump function due to inhibition of ion binding. This implies that D801N substitutions can cause pump dysfunction more than D801Y. Heterozygous knock-out mice and knock-in mice deficient in *ATP1A3* have been generated. The *ATP1A3* knock-out mice were found to have reduced NMDA receptors and exhibited neurological abnormalities such as hyperactivity, spatial learning and memory deficit [19]. The mice harboring mutation I810N of *ATP1A3*, which were neither RPD nor AHC, developed seizures [20]. While these phenotypes do not necessarily correspond with the typical clinical manifestations observed in either RDP or AHC, some similarities do exist.

In total, we identified four *ATP1A3* mutations in 10 Japanese AHC patients. All were heterozygous and *de novo*. Although the number of patients was small (10 individuals), E815K and D801N were observed in 5 (50%) and 3 (30%) of the 10 patients, respectively.

The exact mechanism of *de novo* mutation identified in this study is not clear at present. The nucleotides of both E815K and D801N are located in the GC-rich sequences of *ATP1A3*, and within 6-bp palindrome. These features may be related to the development of these *de novo* mutations.

Intriguingly, E815K mutation of *ATP1A3* found in half of our patients was associated with the presence of severe neurological symptoms, respiratory failure, status epilepticus and resistance to medications. The attending physicians consider, with hindsight clinical experience that flunarizine seems to be less effective in individuals with E815K mutation, compared to those with other mutations. However, the association between genotype and phenotype remains undefined due to the small number of the cohort. The present findings and those of other groups on AHC associated with *ATP1A3* mutations warrant further studies to understand the relation between genotype and phenotype in AHC and to develop new tools for the diagnosis and treatment of AHC.

## Patients and Methods

### Ethics statement

The present study was approved by the Ethics Review Committees of Fukuoka University and the University of Tokyo. Parents of each patient and the parents themselves provided signed informed consent before the study.

### Patients

We initially recruited 10 unrelated Japanese individuals with clinical features of typical sporadic AHC. The diagnosis of AHC was based on the criteria of AHC [1], [2]. The clinical presentations of these patients were typical but the neurological symptoms showed some variations, including aphonia, choreoathetosis, dyskinesia, epilepsy, and episodic apnea. Furthermore, variability in the response to different medications, such as flunarizine, was also noted among the patients (Table 3). Flunarizine was used for the treatment of 9 patients to control paralysis. The frequency of the paretic symptom decreased somewhat following the treatment, compared to that with other medications. However, the response to treatment, as evaluated subjectively by the attending physician, was not remarkable. Two patients (II-1 and V-1) showed a better response to clonazepam than to flunarizine.

The patients studied were 8 males and 2 females with similar clinical presentation, including infantile onset and psychomotor retardation. MRI images showed high-intensity hippocampal region in patient VIII-1 (Table 3), which was considered secondary to repeated episodes of epileptic convulsions. MRI images in patients II-1 and III-1 showed cerebellar atrophy, which was considered a primary lesion similar to FHM. The MRI findings in patient V-1 were considered non-specific.

Based on the availability of samples from the parents of the 9 patients, we selected 8 probands (subjects I-1 to VIII-1, Table 3) for exome sequencing analysis. After the identification of *de novo* heterozygous mutations in 8 patients, we also collected samples from the parents of patient IX-1 and also samples from patient X-1 and his parents. Parents of the patients with available genomic DNAs were also enrolled in this study. We also recruited 96 unrelated healthy Japanese volunteers as the control group who were free of seizures or history of epilepsy.

Genomic DNA was prepared from EDTA-Na_2_-containing blood samples using the QIAamp DNA Blood Maxi Kit (Qiagen, Hilden, Germany), using the protocol provided by the manufacturer.

### Exome sequencing

The exonic sequences were enriched using the Agilent SureSelect technology for targeted exon capture (213,383 exons, covering approximately 50 Mb of the CCDS database) (Agilent Technologies, Santa Clara, CA) from 3 µg of genomic DNA, using the protocol provided by the manufacturer. The captured DNAs were subjected to massively parallel sequencing (100 bp paired-end reads) on the Illumina Hiseq2000 (Illumina, San Diego, CA). The average of 1.3 billion bases of the sequence data was obtained for each individual. On average, 99.08% of the total bases were mapped to the reference genome with a mean coverage of 182.8x, which encompassed 92.99% of the targeted regions with coverage >10x. Burrows Wheeler Aligner [21] and Samtools [22] were used as default settings for alignment of raw reads and detection of variations. The variants were filtered against dbSNP (build 135). The aligned short reads were viewed using the University of Tokyo Genome Browser (UTGB) [23].

### Sanger sequencing

Sanger sequencing was performed to validate the presence of each variant detected by exome sequencing in patients with AHC and the absence of each in the parental genomes. The entire exons and the intron-exon boundaries of *ATP1A3*, *CNTN4* (NM_175607) and *SYNE1* (NM_033071) were amplified by PCR using the designed PCR primers (Table S1 lists the primer sequences and the PCR conditions). The PCR products were purified in ExoSAP-IT for PCR Product Clean-Up (Affymetrix, Santa Clara, CA) set at one cycle of 15 min at 37°C and 15 min at 80°C. The purified PCR products were sequenced using the ABI PRISM BigDye 3.1 terminator method (Applied Biosystems, Foster City, CA) and the ABI PRISM® 3100 Genetic Analyzer (Applied Biosystems).

### URLs

BLAST: http://blast.ncbi.nlm.nih.gov/Blast.cgi?CMD=Web&PAGE_TYPE=BlastHome Japanese Society of Alternating hemiplegia of childhood: http://www008.upp.so-net.ne.jp/ahc/

### Accession numbers

Reference sequences are available from NCBI under the following accession codes: *CACNA1A:*NM_000068

*ATP1A2*:MN_000702

*CNTN4:* NM_175607

*ATP1A3:* NM_152296

*SYNE1:* NM_033071

## Supporting Information

Figure S1 **Rations of single nucleotide variations (SNVs) overlapping with known polymorphisms in various ethnic backgrounds.**
(DOC)

Note S1 **Brain-expressed genes.**
(DOC)

Table S1 **PCR primers and conditions designed for** ***ATP1A3*** **.**
(DOC)
