# Supplementary material for: Identification of ATP1A3 Mutations by Exome Sequencing as the Cause of Alternating Hemiplegia of Childhood in Japanese Patients
Source: PLoS One. 2013 Feb 8;8(2):e56120. doi: 10.1371/journal.pone.0056120 (PMC3568031; doi:10.1371/journal.pone.0056120)
Supplement: Figure S1 — Rations of single nucleotide variations (SNVs) overlapping with known polymorphisms in various ethnic backgrounds. (DOC) [file pone.0056120.s001.doc]

**Figure S1. Rations of single nucleotide variations (SNVs) overlapping with known polymorphisms in various ethnic backgrounds**

**
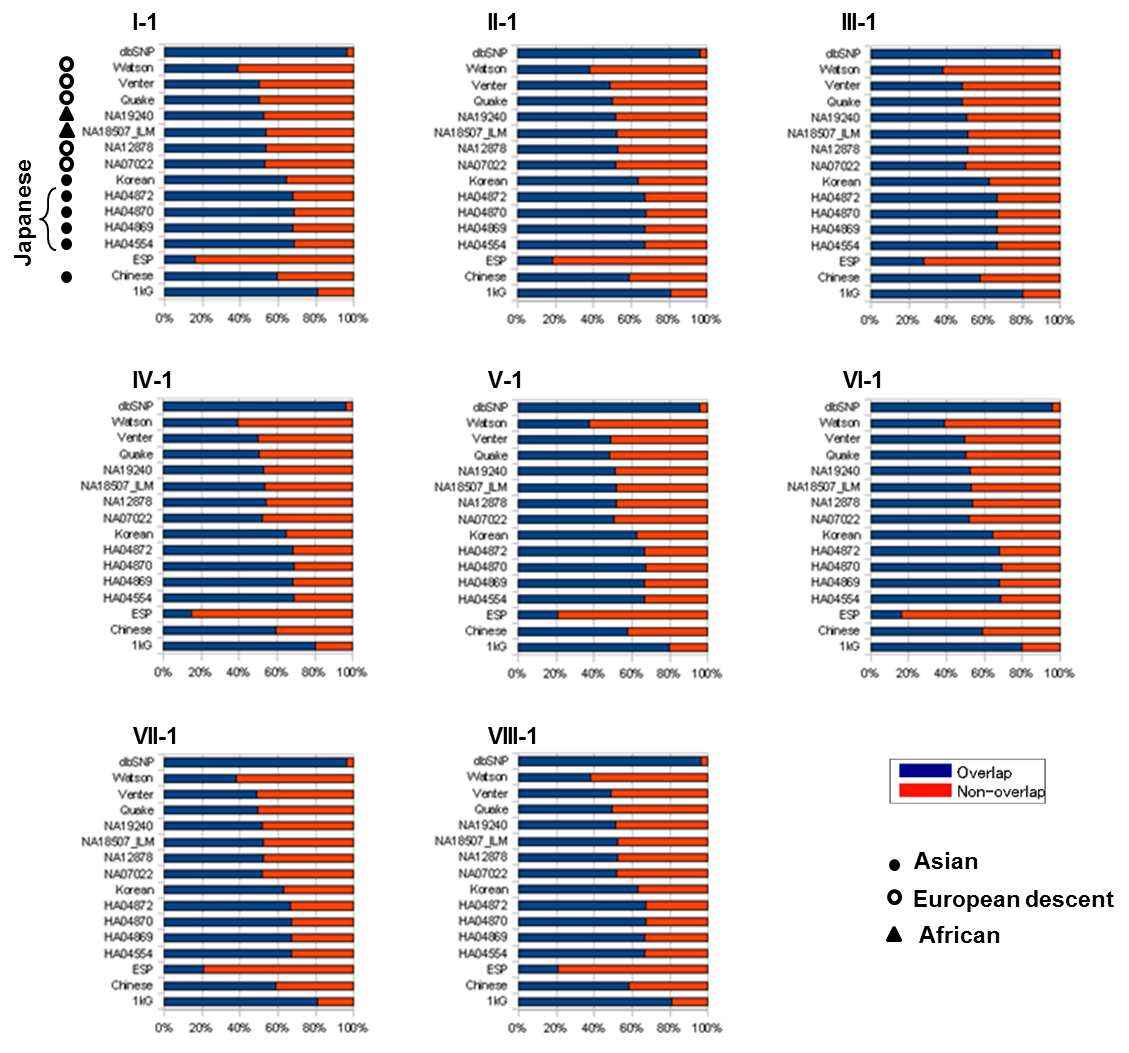
**

SNVs of eight unrelated individuals with AHC were compared with known polymorphisms of dbSNP (http://www.ncbi.nlm.nih.gov/projects/SNP/); 1000 Genomes project (http://www.1000genomes.org/), NHLBI Exome Sequencing Project (ESP: http://evs.gs.washington.edu/EVS/), Personal Genome Project 10 (PGP-10: http://www.personalgenomes.org/pgp10.html), six Asian (including a Chinese [1], a Korean [2] and four Japanese: http://www.1000genomes.org/), five individuals of European descent (Watson [[3](#_ENREF_3)], Venter , Quake and NA12878 & NA07022: http://www.1000genomes.org/) and two Africans (NA19240 & NA18507: http://www.1000genomes.org/). The ratios of non-overlapping variations in these patients are comparable to those of Asian or Japanese populations.

**References**

**1. Wang J, Wang W, Li R, Li Y, Tian G, et al. (2008) The diploid genome sequence of an Asian individual. Nature 456: 60-65.**

**2. Kim JI, Ju YS, Park H, Kim S, Lee S, et al. (2009) A highly annotated whole-genome sequence of a Korean individual. Nature 460: 1011-1015.**

**3. Wheeler DA, Srinivasan M, Egholm M, Shen Y, Chen L, et al. (2008) The complete genome of an individual by massively parallel DNA sequencing. Nature 452: 872-876.**

**4. Levy S, Sutton G, Ng PC, Feuk L, Halpern AL, et al. (2007) The diploid genome sequence of an individual human. PLoS Biol 5: e254.**

**5. Pushkarev D, Neff NF, Quake SR (2009) Single-molecule sequencing of an individual human genome. Nat Biotechnol 27: 847-850.**
