# Supplementary material for: Identification of ATP1A3 Mutations by Exome Sequencing as the Cause of Alternating Hemiplegia of Childhood in Japanese Patients
Source: PLoS One. 2013 Feb 8;8(2):e56120. doi: 10.1371/journal.pone.0056120 (PMC3568031; doi:10.1371/journal.pone.0056120)
Supplement: Note S1 — Brain-expressed genes. (DOC) [file pone.0056120.s002.doc]

**Note S1**

**Brain-expressed genes**

A list of brain-expressed genes was obtained from a recent study of the human brain transcriptome throughout development and adulthood . The 1,340-sample dataset of that study was generated by dissecting regions from 57 clinically unremarkable postmortem brains of donors ranging in age from 6 post conceptual weeks to 82 years (divided into 15 age bins). The expression levels of 17,565 protein-coding genes within each sample were assayed using the Affymetrix Gene Chip Human Exon 1.0 ST Array platform. A “brain-expressed” gene was defined as having a log2-transformed signal intensity ≥6 in at least one sample and a mean detection above background (DABG) P<0.01 in at least one brain region of at least one period. Using these criteria, 630 of 2,131 genes in SNVs and 71 of 232 in indels were expressed in at least one brain region during at least one age bin. Using this list of 630 genes in SNVs and 71 genes in indels, the 2,449 rare SNVs and 246 rare indels in individuals with AHC were defined as being "brain-expressed" (718 and 76, respectively) or not brain-expressed (1,731 and 170, respectively). In text, Table, and Supporting figures and legends describing the results as "brain-expressed", the latter means they were on the list of 701 genes.

**Reference**

**1. Kang HJ, Kawasawa YI, Cheng F, Zhu Y, Xu X, et al. (2011) Spatio-temporal transcriptome of the human brain. Nature 478: 483-489.**
