# Supplementary material for: Identification of ATP1A3 Mutations by Exome Sequencing as the Cause of Alternating Hemiplegia of Childhood in Japanese Patients
Source: PLoS One. 2013 Feb 8;8(2):e56120. doi: 10.1371/journal.pone.0056120 (PMC3568031; doi:10.1371/journal.pone.0056120)
Supplement: Table S1 — PCR primers and conditions designed for ATP1A3 . (DOC) [file pone.0056120.s003.doc]

**Table S1. PCR primers and conditions designed for *ATP1A3.***

| Exon number | primer sequence (5'→3') | | PCR product (bp) | Annealing temperature (°C) |
| --- | --- | --- | --- | --- |
| Exon1 | F | GCCACTTTGCGGAGCCCAAG | 361 | 68 |
|  | R | CCGCGCACACCCAATGTCAC |  |  |
| Exon2-3 | F | AGTCACCAGAGGCAGAAGGGAGGTGAAGTC | 513 | 60 |
|  | R | TCCTAGCCCCCAGCTCCTCCTCCCTAGTTC |  |  |
| Exon4 | F | GCTGGTGTCTTAAGTTCTGG | 365 | 60 |
|  | R | TGAAAAGCTTAGAGGGATGG |  |  |
| Exon5 | F | AACCCTCATAACCAACCTAG | 350 | 60 |
|  | R | GTGGGATGGAGTAATGTCAC |  |  |
| Exon6 | F | GTGAAGGGTGCCCAGCAAGG | 336 | 68 |
|  | R | AGGGGTTGGGACCTGGACTC |  |  |
| Exon7 | F | GTGATCTCCAGGCACACAG | 317 | 60 |
|  | R | AGGCCAGAGGGGTTAGGCTG |  |  |
| Exon8 | F | GAAGGTGAGGCGGGTGCAGAGAAGACACAC | 494 | 68 |
|  | R | TCCCTCAGACTCAGGGGTCCAGGATCCCAG |  |  |
| Exon9 | F | TTTCCAGGCCCTCAGGATTC | 363 | 60 |
|  | R | TGTGTGAGGGCCAGGGACTC |  |  |
| Exon10 | F | GACACCACTGAGGACCAGTC | 337 | 60 |
|  | R | GCCAGAGGACAGCTCGATGC |  |  |
| Exon11 | F | GGTTCAGCTACTGGCCTCAC | 358 | 60 |
|  | R | TCCGGCTCCCACTCTCAAGC |  |  |
| Exon12 | F | GAGGTCTCTGTGAGAGAGTG | 373 | 60 |
|  | R | AGCATCACAACCCTCCTTGC |  |  |
| Exon13 | F | GAGATGGAATGCGGGCGATG | 341 | 60 |
|  | R | GTGACCATGATGACCTGCAG |  |  |
| Exon14 | F | TGACGCGGTGGGCAAGTGTC | 361 | 60 |
|  | R | GCCTGGTCCATGGAGGAGTC |  |  |
| Exon15 | F | CAGGGATCACTTTGCCACTC | 332 | 60 |
|  | R | CAGACCCTCCTCTCTCAGAC |  |  |
| Exon16 | F | GTCCTGGGCTTCTGGATCTG | 300 | 60 |
|  | R | CTCAGGCCTCCGGTAGTGAC |  |  |
| Exon17 | F | GACGTTGGATGAGGGCAGAG | 326 | 60 |
|  | R | CCTCGTACGCCAGTGAGATG |  |  |
| Exon18 | F | CTGCATCGATCTGGGCACTGACATGGTGAG | 376 | 68 |
|  | R | AGACCCCCCCCACAGATAGCTCACTGGTTG |  |  |
| Exon19 | F | ATACTCCCCTCTCCAAGGAG | 317 | 60 |
|  | R | CATCGTAGGAAGTGGCCATG |  |  |
| Exon20 | F | ATGAGCCCGCAGAAGGAAGACACACCTGAG | 405 | 68 |
|  | R | CAGAGTGAGACCCTGCCTCAACAAAACAAC |  |  |
| Exon21 | F | CTCTGGGTGTCTGCACTGTG | 338 | 60 |
|  | R | GTGGTGGGAGCAGCCTATGG |  |  |
| Exon22 | F | CGAAGGATCCTGGGAGACTG | 339 | 60 |
|  | R | ACCCGTGAGAAGACAGAGTG |  |  |
| Exon23 | F | GTCTGTGTGGTTTCCTTGTCTCTCTCCCTC | 541 | 60 |
|  | R | CAGTCCCCGTCTGGAAAGCAGGCACAACAC |  |  |
